# Cytokine adsorption in patients with acute-on-chronic liver failure (CYTOHEP)—a single center, open-label, three-arm, randomized, controlled intervention trial

**DOI:** 10.1186/s13063-022-06139-6

**Published:** 2022-03-18

**Authors:** Asieb Sekandarzad, Enya Weber, Eric Peter Prager, Erika Graf, Dominik Bettinger, Tobias Wengenmayer, Alexander Supady

**Affiliations:** 1 Department of Medicine III (Interdisciplinary Medical Intensive Care), Medical Center – University of Freiburg, Faculty of Medicine, University of Freiburg, Freiburg, Germany; 2 Department of Cardiology and Angiology I, Heart Center, University of Freiburg, Freiburg, Germany; 3 Institute of Medical Biometry and Statistics, Faculty of Medicine and Medical Center – University of Freiburg, Freiburg, Germany; 4 Department of Medicine IV (Nephrology and General Medicine), Medical Center – University of Freiburg, Faculty of Medicine, University of Freiburg, Freiburg, Germany; 5 Department of Medicine II (Gastroenterology), Medical Center – University of Freiburg, Faculty of Medicine, University of Freiburg, Freiburg, Germany; 6 Heidelberg Institute of Global Health, University of Heidelberg, Heidelberg, Germany

**Keywords:** Liver cirrhosis, Acute-on-chronic liver failure, Extracorporeal hemoadsorption, CytoSorb, Randomized controlled trial

## Abstract

**Background:**

Liver cirrhosis is a major healthcare problem and the mortality rate is high. During recent years, systemic inflammation has been recognized as a major driver of hepatic decompensation and progression of liver cirrhosis to acute-on-chronic liver failure (ACLF). The aim of the CYTOHEP study is to assess the impact of extracorporeal hemoadsorption with the CytoSorb adsorber on serum bilirubin concentrations, humoral inflammation parameters, liver function parameters, and patient survival in patients with ACLF and acute kidney injury (AKI).

**Methods:**

The CYTOHEP study is a prospective, single-center, open-label, three-arm, randomized, controlled intervention trial. Patients with ACLF and AKI stage 3 according to Kidney Disease: Improving Global Outcome (KDIGO) criteria will be randomized into three groups to be treated with (1) continuous renal replacement therapy (CRRT) and CytoSorb, (2) CRRT without CytoSorb, and (3) without both, CRRT and CytoSorb. In the hemoadsorption group, CytoSorb will be used for 72 h. The other groups receive standard of care with early or late initiation of CRRT, respectively. Primary endpoint of the study is serum bilirubin concentration after 72 h, important secondary endpoints are 30-day survival and a panel of inflammatory parameters.

**Discussion:**

The CYTOHEP study is designed to evaluate the benefit of extracorporeal hemoadsorption in patients with ACLF. The results of this study will help to better understand the potential role of hemoadsorption for the treatment of ACLF and its impact on bilirubin levels, inflammatory parameters, and survival.

**Trial registration:**

ClinicalTrials.gov NCT05019352. Registered on August 24, 2021. Deutsches Register Klinischer Studien (DRKS) DRKS00026082.

## Clinical Trial Protocol

**Table:** 

1. **Administrative Information**	
1.1. **Title**	
Cytokine adsorption in patients with acute-on-chronic liver failure (CYTOHEP)—a single center, open-label, three-arm, randomized, controlled intervention trial	
1.2. **Trial registration**	
1.2.1. **Trial registry name and trial identifier**	
ClinicalTrials.gov: NCT05019352	
Deutsches Register Klinischer Studien (DRKS): DRKS00026082	
1.2.2. **Synopsis/World Health Organization Trial Registration Data Set**	
**Data category**	**Information**
Primary registry and trial identifying number	ClinicalTrials.gov: NCT05019352
Date of registration in primary registry	August 24, 2021
Secondary identifying numbers	DRKS00026082
Source(s) of monetary of material support	University of Freiburg, Medical Center, Department of Medicine III, Intensive Care Medicine, Faculty of Medicine, University of Freiburg
Primary sponsor	University of Freiburg, Medical Center, Department of Medicine III - Intensive Care Medicine
Secondary sponsor(s)	None
Contact for public queries	University of Freiburg
Dr. Alexander Supady, MPH	Medical Center Department of Medicine III - Intensive Care Medicine Hugstetter Str. 55 79106 Freiburg Germany Tel.: +49 761 270-73790 Fax: +49 761 270-73792 E-mail: alexander.supady@uniklinik-freiburg.de
Contact for scientific queries Dr. Alexander Supady, MPH	University of Freiburg Medical Center Department of Medicine III - Intensive Care Medicine Hugstetter Str. 55 79106 Freiburg Germany Tel.: +49 761 270-73790 Fax: +49 761 270-73792 E-mail: alexander.supady@uniklinik-freiburg.de
Public title	Cyctokine adsorption in acute-on-chronic liver failure (CYTOHEP trial)
Scientific title	Cytokine adsorption in patients with acute-on chronic liver failure (CYTOHEP) – a single center, open-label, three-arm, randomized, controlled intervention trial
Countries of recruitment	Germany
Health condition(s) or problem(s) studied	Liver cirrhosis; acute-on-chronic liver failure (ACLF)
Intervention(s)	Active comparator: continuous renal replacement therapy with cytokine adsorption for 72 h control comparator: continuous renal replacement therapy without cytokine adsorption control comparator: no continuous renal replacement therapy and no cytokine adsorption
Key inclusion and exclusion	Inclusion criteria • Adult patients (≥ 18 years) admitted to the University Medical Center Freiburg, Germany • Acute-on-chronic liver failure (ACLF) [1] **WITH** o Acute kidney injury according to Kidney Disease: Improving Global Outcome (KDIGO) criteria stage 3 (≥ 3-fold increase of serum creatine **OR** increase of serum creatine ≥ 4 mg/dl **OR** urine output ≤ 0.3 ml/kg/h for ≥ 24 h **OR** anuria for ≥ 12 h) **AND** o Serum bilirubin ≥ 5 mg/dl Exclusion criteria • Known patient will against participation in the study or against the measures applied in the study • A decision made prior to inclusion to stop further treatment of the patient within the next 24 h • No complete remission of malignancy including hepatocellular carcinoma within the past 12 months • Patients on the waiting list for liver transplant or the potential option for being listed for liver transplant within the next 6 months • Liver cirrhosis in patients after liver transplantation • Ongoing intermittent or continuous renal replacement therapy before study inclusion All patients that fulfill all inclusion criteria and none of the exclusion criteria, will be considered *provisionally eligible*. In a subsequent assessment it will be ascertained whether the most responsible clinician(s) (the attending critical care physician and where relevant, the attending nephrologist) are in a position of clinical equipoise with respect to the two renal-replacement therapy initiation strategies that the provisionally eligible patient would receive if he/she was randomized. This will be performed in practice by ascertaining the presence of the following two exclusion criteria: • Clinician(s) caring for the patient believe that immediate renal-replacement therapy is mandated. After fulfilling the above inclusion/exclusion criteria, the study team has to speak to the ICU and/or nephrology attending physician and ask if he/she agrees with the statement: “Renal-replacement therapy must be initiated immediately for this patient.” If the answer is “Yes”, the patient will be excluded but could be re-screened for eligibility, if applicable. • Clinician(s) caring for the patient believe that deferral of renal-replacement therapy is mandated. After fulfilling the above inclusion/exclusion criteria, the study team has to speak to the ICU and/or nephrology attending physician and ask if he/she agrees with the statement: “Renal-replacement therapy must be deferred for this patient.” If the answer is “Yes”, the patient will be excluded, but could be re-screened for eligibility. When on both questions above it will be replied “No” by all of the relevant clinicians, the respective patient will be considered fully eligible for study participation.
Study type	Interventional Allocation: randomized; Intervention model: parallel assignment; Masking: open-label Primary purpose: treatment of severe acute-on-chronic liver failure Post-approval/pivotal study
Date of first enrollment	December 12, 2021
Target sample size	51
Recruitment status	Recruitment phase
Primary outcome(s)	Serum bilirubin reduction after 72 h
Key secondary outcomes	• Survival time (days) from baseline • Interleukin-6 after 72 h • Liver function parameters (72 h): Quick/INR, AST, ALT, AP, g-GT • Blood lactate (72 h) • Clinical scores: CLIF-SOFA-score, MELD score, SOFA score, SAPS II and FIPS score (72 h) • Ventilator free days (VeFD) in the first 30 days after randomization, where each day on invasive mechanical ventilation (IMV), non-invasive ventilation (NIV), or ECMO is defined as ventilator day. VeFD=0, if the patient dies in the first 30 days after randomization • Vasopressor free days (VaFD) in the first 30 days after randomization, where each day with any dose of epinephrine, norepinephrine, dobutamine, argipressin or terlipressin is defined as vasopressor day. VaFD=0, if the patient dies in the first 30 days after randomization • Dialysis free days (DFD) in the first 30 days after randomization, where each day on renal replacement therapy (RRT) is defined as dialysis day. DFD=0 if the patient dies in the first 30 days after randomization • A biomarker panel of pro- and anti-inflammatory cytokines (blood samples will be frozen and stored for later analyses, panel will be determined at the time of analysis)
1.3. **Protocol version**	
Version 1.3, 27.01.2022 Revision chronology:	
	• Version 1.0; submitted to IRB for approval (02.07.2021); revision requested by IRB (05.08.2021) • Version 1.1; revised version, considering previous request by IRB (09.08.2021); IRB approval (17.08.2021) • Version 1.2.; amendment submitted to IRB (04.10.2021); addition of trial registration information, specification of randomization tool; IRB approval (19.10.2021)
1.4. **Trial status** Recruitment phase. Recruiting started December 12, 2021. Estimated trial completion by December 31, 2023. 1.5. **Protocol reporting guidelines** This clinical trial protocol is following SPIRIT reporting guidelines (see checklist in the supplement) [1]. 1.6. **Funding** The study is financed by internal funds from the University of Freiburg Medical Center, Department of Medicine III - Intensive Care Medicine. A grant from the Faculty of Medicine, Freiburg University within the funding program for clinical trials will be applied for. 1.7. **Roles and responsibilities** ASe, ASu and DB designed the trial and wrote the first draft of the study protocol. EW and EG performed the sample size estimation and set up and wrote the statistical analysis plan. EP advised for specifics related to renal replacement therapy. EP and TW revised the manuscript and added important content. All co-authors reviewed and approved the final version of the manuscript. 1.7.1. **Sponsor-investigator/Principal Investigator** Dr. Alexander Supady, MPH University of Freiburg Medical Center Department of Medicine III - Intensive Care Medicine Hugstetter Str. 55 79106 Freiburg Germany Tel.: +49 761 270-73790 Fax: +49 761 270-73792 E-mail: alexander.supady@uniklinik-freiburg.de 1.7.2. **Co-Principal Investigator** PD Dr. Dominik Bettinger University of Freiburg Medical Center Department of Medicine II Hugstetter Str. 55 79106 Freiburg Germany Tel.: +49 761 270-34010 or -32440 E-mail: dominik.bettinger@uniklinik-freiburg.de 1.7.3. **Co-Principal Investigator** Dr. Asieb Sekandarzad University of Freiburg Medical Center Department of Medicine III - Intensive Care Medicine Hugstetter Str. 55 79106 Freiburg Germany Tel.: +49 761 270-33322 E-mail: asieb.sekandarzad@uniklinik-freiburg.de 1.7.4. **Cooperating investigator** Dr. Eric Peter Prager University of Freiburg Medical Center Department of Medicine IV Hugstetter Str. 55 79106 Freiburg Germany Tel.: +49 761 270-34140 E-mail: eric.peter.prager@uniklinik-freiburg.de 1.7.5. **Trial statistician** Dr. Erika Graf University of Freiburg Institute of Medical Biometry and Statistics (IMBI) Stefan-Meier-Straße 26 79104 Freiburg Germany Tel.: +49 761 270-83743 E-mail: erika.graf@uniklinik-freiburg.de 1.7.6. **Role of study sponsor and funders** The study will be financed from internal funds from the University of Freiburg, Medical Center, Department of Medicine III - Intensive Care Medicine and the University of Freiburg Faculty of Medicine. The sponsor-investigator/principal investigator is an employee of University of Freiburg Medical Center. The sponsor-investigator/principal investigator is independently responsible for collection, management, analysis, and interpretation of data, writing of the report(s) and the decision to submit the report for publication.	

## Introduction

### Background and rationale

#### Systemic inflammation in decompensated liver cirrhosis and acute-on-chronic liver failure (ACLF)

Liver cirrhosis is a major healthcare problem. Among affected patients mortality is high. In Germany, in 2015 more than 13,000 patients died owing to decompensated cirrhosis [2]. The clinical course of cirrhosis can be separated in compensated and decompensated cirrhosis. Patients with compensated cirrhosis are largely asymptomatic and the development of decompensating events is a major hallmark in the course of the disease as median survival decreases from 12 years to less than 2 years [3]**.** The most important decompensating events are the development of variceal bleeding, ascites, hepatic encephalopathy, and bacterial infections [4]. Previously, liver cirrhosis has been described as a systemic disease affecting almost any other organ system [4, 5]. The development of extrahepatic organ complications in decompensated cirrhosis has been identified as a major prognostic milestone and has been described as acute-on-chronic liver failure (ACLF) [6]. ACLF is understood as a dynamic process and may evolve within days leading to multi-organ failure with renal failure being the most common organ involvement (56%), followed by liver and coagulation failure (44% and 28%, respectively) [6, 7]. ACLF is associated with a high 28-day mortality ranging from 22% to 77% in patients with multi-organ failure [6].

During recent years, systemic inflammation has been recognized as a major driver of hepatic decompensation and progression of liver cirrhosis to ACLF. Systemic inflammation steadily increases with the progression of decompensated liver cirrhosis and the development of ACLF [4, 8]. Importantly, systemic inflammation was described as an important trigger for the development of extrahepatic organ failures, such as renal failure, development of hepatopulmonary syndrome, cirrhotic cardiomyopathy, and hepatic encephalopathy [4, 9, 10]. The evolution of systemic inflammation in patients with progressing hepatic decompensation and ACLF is due to an increase in bacterial translocation in the intestine [4, 11]. Bacterial translocation is associated with portal hypertension which is one of the major drivers of decompensation in cirrhotic patients and determines a hallmark in the course of disease [4, 12]. Damage-associated molecular patterns (DAMPs) and pathogen-associated molecular patterns (PAMPs) may serve as a surrogate for bacterial translocation in patients with liver cirrhosis [4]. In summary, systemic inflammation is particularly relevant in the pathogenesis of acute hepatic decompensation and is also associated with reduced survival [13]. Therefore, elimination of PAMPs, DAMPs, and inflammatory cytokines in addition to established therapeutic approaches aiming at a reduction of bacterial translocation and mitigation of portal hypertension may help control excessive inflammatory activity and thus support hepatic recompensation. This may ultimately lead to improved survival.

Previous in-vitro examinations and studies in non-cirrhotic inflammatory disorders have shown that proinflammatory cytokines, DAMPs, and PAMPs can effectively be removed by extracorporeal hemoadsorption in the CytoSorb adsorber [14].

#### CytoSorb cytokine adsorber

The CytoSorb adsorber (CytoSorbents Corporation, Monmouth Junction, NJ, USA) is a medical device approved for the removal of cytokines, bilirubin, and myoglobin by hemoadsorption. The adsorber can be installed in any kind of extracorporeal blood circuit, such as extracorporeal membrane oxygenation (ECMO) or continuous renal replacement therapy (CRRT), via an associated tubing and connector system using Luer-Lock connections.

The adsorber consists of a cylindrical cartridge filled with tiny, highly porous, biocompatible, and hemocompatible polyvinyl-pyrrolidone-coated polystyrene-divinyl-benzene polymer beads with a total surface area of > 40,000 m^2^ per adsorber [15]. The polymer adsorbs hydrophobic molecules within the 5–55 kDa range; these include, among others, bilirubin, and vasoactive cytokines, associated with acute decompensation in ACLF [4, 15]. Within the device, molecules are adsorbed based on their physicochemical properties (molecular weight, size, solvation tendency) independent of physiological or pathophysiological functions. Therefore, not only harmful substances, but also pathophysiologically “necessary” or “desired” substances, e.g., anti-inflammatory cytokines or drugs, may be adsorbed. The adsorption capacity is postulated to be concentration-dependent, so that low physiological levels are not significantly affected [16].

#### Rationale for the study design

The CYTOHEP study is designed as a prospective, randomized, single-center, open-label, controlled intervention trial to assess the benefit of extracorporeal hemoadsorption using the CytoSorb device in patients with acute-on-chronic liver failure. The primary goal for this trial is to assess whether the CytoSorb device used in addition to CRRT will be able to significantly reduce bilirubin in the patient blood as compared to the control group treated with CRRT alone (i.e., without extracorporeal hemoadsorption).

Within this trial, CRRT will be initiated early, i.e., in patients with acute kidney injury (AKI) Kidney Disease: Improving Global Outcome (KDIGO) stage 3 (see the “Subject inclusion criteria,” “Subject exclusion criteria,” and “Description of study plan and interventions” section for details). For safety assessment, a third group will be assessed without early initiation of CRRT and extracorporeal hemoadsorption. After trial inclusion, all patients will be randomized in a 1:1:1 fashion in one of the study groups.

The rationale for this study is based on considerations about the role of systemic inflammation in acute decompensation of liver cirrhosis and ACLF (see the “Specific objectives” section), in-vitro data comparing the effectiveness of MARS (molecule adsorbent recirculating system) and CytoSorb for the removal of molecules with a pathophysiological role in acute-on-chronic liver failure, and recent reports on the successful use of extracorporeal hemoadsorption in combination with CRRT in critically ill patients with acute liver dysfunction [17–21]. The choice of the primary endpoint bilirubin reduction after 72 h is based on results from these reports describing the successful elimination of bilirubin with CytoSorb. Additionally, a broad array of clinical and inflammatory parameters will be assessed to better understand the mode of action and molecular effects of the CytoSorb adsorber. The trial is planned to be followed by a subsequent multi-center trial.

#### Risks and benefits

##### Potential risks and benefits associated with the study procedures

Besides study-specific interventions as outlined in this protocol, patients in all study groups, CRRT with CytoSorb, CRRT without CytoSorb, and no CRRT, will be treated according to establish standard of care (SOC), and current guidelines, treatment standards, and recommendations will apply. In addition to SOC treatment, patients in both the CRRT with the CytoSorb group and the CRRT without the CytoSorb group will be treated with continuous renal replacement therapy (CRRT). Patients in the CRRT with CytoSorb group will receive extracorporeal hemoadsorption for a total duration of 72 h. The adsorber will be included in the CRRT machine. For patients in the no CRRT group the use of CRRT will be delayed until CRRT will be otherwise indicated by the treating physicians or one or more of the following criteria will evolve: a serum potassium level of 6.0 mmol or more per liter, a pH of 7.20 or less due to metabolic acidosis or a serum bicarbonate level of 12 mmol per liter or less, evidence of severe respiratory failure based on a ratio of the partial pressure of arterial oxygen to the fraction of inspired oxygen of 200 or less and clinical perception of volume overload.

Within this trial, both in the CRRT with CytoSorb group and in the CRRT without CytoSorb group CRRT will be initiated in patients with ACLF and AKI KDIGO stage 3. The optimal timing of initiation of CRRT in patients with acute kidney injury (AKI) is a matter of debate. Generally, a rather restrictive strategy for initiation of hemodialysis is recommended, i.e., initiating CRRT when life-threatening deterioration in fluid, electrolyte, or acid-base balance evolves that cannot be managed by conservative treatment [22]. Several randomized controlled trials (RCTs) and meta-analyses have compared early versus delayed initiation strategies for CRRT with, in part, conflicting results. One single-center RCT showed a mortality benefit (90 days) for early initiation of CRRT in critically ill patients, i.e., initiation of CRRT when reaching AKI KDIGO stage 2 compared to initiation of CRRT not before reaching AKI KDIGO stage 3 (39.3% vs. 54.7%, *p*=0.03) [23]. A subsequent multicenter RCT, however, did not show a difference in mortality at 60 days when comparing initiation of CRRT in AKI KDIGO stage 3 to an even more restrictive strategy (48.5% vs. 49.7%), i.e., initiation only when potentially life-threatening complications (severe hyperkalemia, metabolic acidosis, pulmonary edema) occurred or a blood urea nitrogen (BUN) level higher than 112 mg per deciliter or oliguria for more than 72 h were present. In this trial, adverse events were similar in both groups, besides more frequent occurrence hypophosphatemia and catheter-related blood stream infections in the early-initiation group (hypophosphatemia: 22% vs. 15%, *p*=0.03, infections: 10% vs. 5%, *p*=0.03) [24].

Recently, another multicenter RCT did not reveal a difference in mortality at day 90 (43.9% vs. 43.7%) when comparing an early initiation strategy of CRRT (i.e., within 12 h after reaching AKI KDIGO stage 2 or 3) with a delayed initiation strategy (i.e., when life-threatening complications of AKI occurred, or AKI stage 2 or 3 persisted for more than 72 h). However, adverse events occurred more frequently in the early initiation group (23% vs. 16.5%, *p*<0.001) and more patients in this group remained dependent on CRRT at 90 days (10.4% vs. 6%, relative risk: 1.74; 95% CI, 1.24 to 2.43) [25].

In a meta-analysis of nine studies comparing early vs. late CRRT initiation strategies in 1879 patients, no difference in survival at 28 days was found (44% vs. 43%) [26].

Finally, another RCT showed that further delaying CRRT in patients with oliguria for more than 72 h or BUN > 112 mg/dl even without life-threatening complications was not beneficial but even associated with an increased 60-day mortality risk (HR=1.65; 95% CI 1.09–2.50, *p*=0.018) [27].

So far, the above-described strategies for initiation of CRRT have not been assessed in patients with ACLF and the results from these trials cannot easily be extrapolated to the patient cohort eligible for this trial. Therefore, in patients eligible for the CYTOHEP study, the optimal timing for initiation of CRRT remains unclear, so far [28, 29].

At least some of the patients eligible for study participation in the CYTOHEP trial will suffer from coagulation disorders due to liver function failure and standard anticoagulation strategy for CRRT with unfractionated heparin can be difficult. On the other hand, citrate anticoagulation, that is usually used in patients with coagulation abnormalities, can rarely cause metabolic acidosis in patients with liver disease as citrate can accumulate in patients with severe hepatic metabolic abnormalities.

Considering the above summarized evidence, we consider initiation of CRRT in patients with AKI KDIGO stage 3 within this trial reasonable and safe. In order to be able to thoroughly assess the safety of the study interventions within this trial, a third group without CRRT will be assessed.

In addition to routine diagnostics, additional blood samples are necessary within the scope of the study at 0 h/baseline/screening and 72 h (43.4 ml each). In the CRRT with CytoSorb group and the CRRT without CytoSorb group, the blood samples are taken via an arterial catheter (A. radialis or A. brachialis) or a central venous catheter, which are necessary for regular therapy monitoring, drug treatment, and renal replacement therapy—no additional vascular punctures are required. Not all patients in the no CRRT group will require an arterial catheter or a central venous catheter. In these patients, blood draws are scheduled to be combined with routine blood draws necessary for treatment guidance, so that no additional vascular punctures are required.

##### Potential risks and benefits associated with the CytoSorb device

A potential concern with the use of the CytoSorb device in patients eligible for this study is the adsorption of drugs, most specifically antibiotics required for the treatment of the patients with ACLF. According to current data and knowledge, penicillins, including combination drugs ampicillin/sulbactam or piperacillin/tazobactam, or cephalosporines, which could be required in this patient population in the case of bacterial infection, are not removed from the patient’s blood to a clinically relevant extent [30, 31]. If the administration of a drug within the adsorption spectrum of the CytoSorb adsorber is necessary for a patient in the CRRT with CytoSorb group and no equivalent alternative is available, the therapy with the CytoSorb adsorber can be terminated at any time.

In a randomized controlled pilot trial in 34 COVID-19 patients on ECMO (CYCOV study), an association between cytokine adsorption and increased mortality has been described [32]. In contrast to these observations, in various retrospective analyses, the use of CytoSorb was described as safe and positive effects have been reported [33–37]. Based on the above-described inflammation hypothesis as a driver of acute decompensation in liver cirrhosis and the observed association between inflammation and mortality in these patients, we postulate a beneficial effect of cytokine adsorption by mitigation of increased inflammatory processes and cascades. However, respecting the results from the CYCOV study, we will continuously monitor and assess the safety of the intervention and closely monitor clinical courses and mortality in all study groups (see also the “Monitoring” and “Early termination of the study” section).

Patients eligible for this study have limited therapeutic options and mortality is high. Based on pathophysiological considerations and preliminary experience using the CytoSorb device in these patients we postulate a positive benefit-risk ratio for the intervention.

#### Study aim and choice of comparator

The aim of the CYTOHEP study is to investigate the influence of cytokine adsorption with the CytoSorb adsorber in combination with CRRT on serum bilirubin concentrations, humoral inflammation parameters, liver function parameters, and patient survival under controlled conditions in patients with ACLF. Since there is equipoise for the use of extracorporeal cytokine adsorption with CRRT in these patients, we directly compare three different treatment strategies, CRRT with CytoSorb, CRRT without CytoSorb, and no CRRT, under otherwise equal conditions and treatment standards and regimens.

Extracorporeal hemoadsorption with the CytoSorb device has to be incorporated into an extracorporeal blood circuit, such as a CRRT circuit. As described above (“Potential risks and benefits associated with the study procedures” section), the evidence as to when to initiate CRRT in the patients eligible for this study (see the “Subject inclusion criteria,” “Subject exclusion criteria” sections for inclusion and exclusion criteria) is incomplete, and therefore, the optimal timing for initiation of CRRT remains unclear, so far. For additional safety within this trial in order to be able to detect early warning signs of a negative effect of early CRRT or CytoSorb compared to a delayed strategy of initiation of CRRT, we included a third study group, the no CRRT group. In this group, patients will be treated without CRRT and without CytoSorb. If indicated, CRRT will be initiated late and only when life-threatening complications occur (see the “Potential risks and benefits associated with the study procedures” section). This approach has been tested in a recent RCT and was not inferior to the approach for early initiation of CRRT as applied in the CRRT with CytoSorb and CRRT without CytoSorb groups [25].

### Objectives

#### Specific objectives

The aim of this study is to show under controlled and randomized conditions that extracorporeal hemoadsorption of bilirubin combined with early initiation of CRRT in patients with ACLF is efficient and safe (CRRT plus CytoSorb versus CRRT without CytoSorb). For safety considerations, a third group will be assessed without early initiation of CRRT and extracorporeal hemoadsorption to generate pilot data on potential negative consequences of early initiation of CRRT in patients with ACLF (CRRT without CytoSorb versus no CRRT).

Furthermore, the trial is designed for an exploratory analysis of a broad array of clinical and inflammatory parameters, including pro- and anti-inflammatory cytokines, with a potential role in ACLF in order to better understand molecular effects and modes of action of the CytoSorb device.

#### Hypothesis

Extracorporeal hemoadsorption using the CytoSorb adsorber in combination with CRRT in patients with ACLF is a safe and efficient method to reduce bilirubin and inflammatory parameters from the patients’ blood and improve the probability of survival.

### Trial design

1:1:1 randomized, controlled, parallel-group, single-center open-label superiority trial.

## Methods

### Participants, interventions and outcomes

#### Study setting

This single-center trial will be conducted at the University Medical Center Freiburg, an academic tertiary care center.

#### Eligibility criteria

##### Subject inclusion criteria

- Adult patients (≥ 18 years) admitted to the Freiburg University Medical Center, Germany
- Acute-on-chronic liver failure (ACLF) [6] WITH
  - Acute kidney injury according to Kidney Disease: Improving Global Outcome (KDIGO) criteria stage 3 (≥ 3-fold increase of serum creatinine OR increase of serum creatinine to ≥ 4 mg/dl OR urine output ≤ 0.3 ml/kg/h for ≥ 24 h OR anuria for ≥ 12 h) AND
  - Serum bilirubin ≥ 5 mg/dl

##### Subject exclusion criteria

- Known patients will be against participation in the study or against the measures applied in the study
- A decision made prior to inclusion to stop further treatment of the patient within the next 24 h
- No complete remission of malignancy including hepatocellular carcinoma within the past 12 months
- Patients on the waiting list for liver transplant or the potential option for being listed for liver transplant within the next 6 months
- Liver cirrhosis in patients after liver transplantation
- Ongoing intermittent or continuous renal replacement therapy before study inclusion

According to the approach used in the STARRT-AKI trial, all patients that fulfill all inclusion criteria and none of the exclusion criteria will be considered provisionally eligible [25]. In a subsequent assessment, it will be ascertained whether the most responsible clinician(s) (the attending critical care physician and where relevant, the attending nephrologist) are in a position of clinical equipoise with respect to the two renal-replacement therapy initiation strategies that the provisionally eligible patient would receive if he/she was randomized. This will be performed in practice by ascertaining the presence of the following two exclusion criteria:

- Clinician(s) caring for the patient believe that immediate renal-replacement therapy is mandated. After fulfilling the above inclusion/exclusion criteria, the study team has to speak to the ICU and/or nephrology attending physician and ask if he/she agrees with the statement: “Renal-replacement therapy must be initiated immediately for this patient.” If the answer is “Yes”, the patient will be excluded but could be re-screened for eligibility, if applicable.
- Clinician(s) caring for the patient believe that deferral of renal-replacement therapy is mandated. After fulfilling the above inclusion/exclusion criteria, the study team has to speak to the ICU and/or nephrology attending physician and ask if he/she agrees with the statement: “Renal-replacement therapy must be deferred for this patient.” If the answer is “Yes”, the patient will be excluded, but could be re-screened for eligibility.

When on both questions above it will be replied “No” by all of the relevant clinicians, the respective patient will be considered fully eligible for study participation.

##### Subject withdrawal criteria

The only reason for the withdrawal of a participant from the study will be withdrawal of informed consent by the participant or a legally authorized representative for any reason. All efforts should be made to ensure the completion of the withdrawal procedures and assessments for patients who discontinue the study early. A request to continue the collection of safety information will be made of all patients who withdraw from the study early and consent for the data collection will be documented.

##### Individuals performing the intervention

Continuous renal replacement machines (CRRT) will be set up and connected to the patient blood circuit by experienced nurses. Exchange of the CytoSorb adsorbers in the CRRT system will also be performed by experienced staff specifically trained for this procedure. There is a 24/7 on-call service for hemodialysis nurses in our hospital.

#### Interventions

##### Description of study plan and interventions

**Fig. 1:**
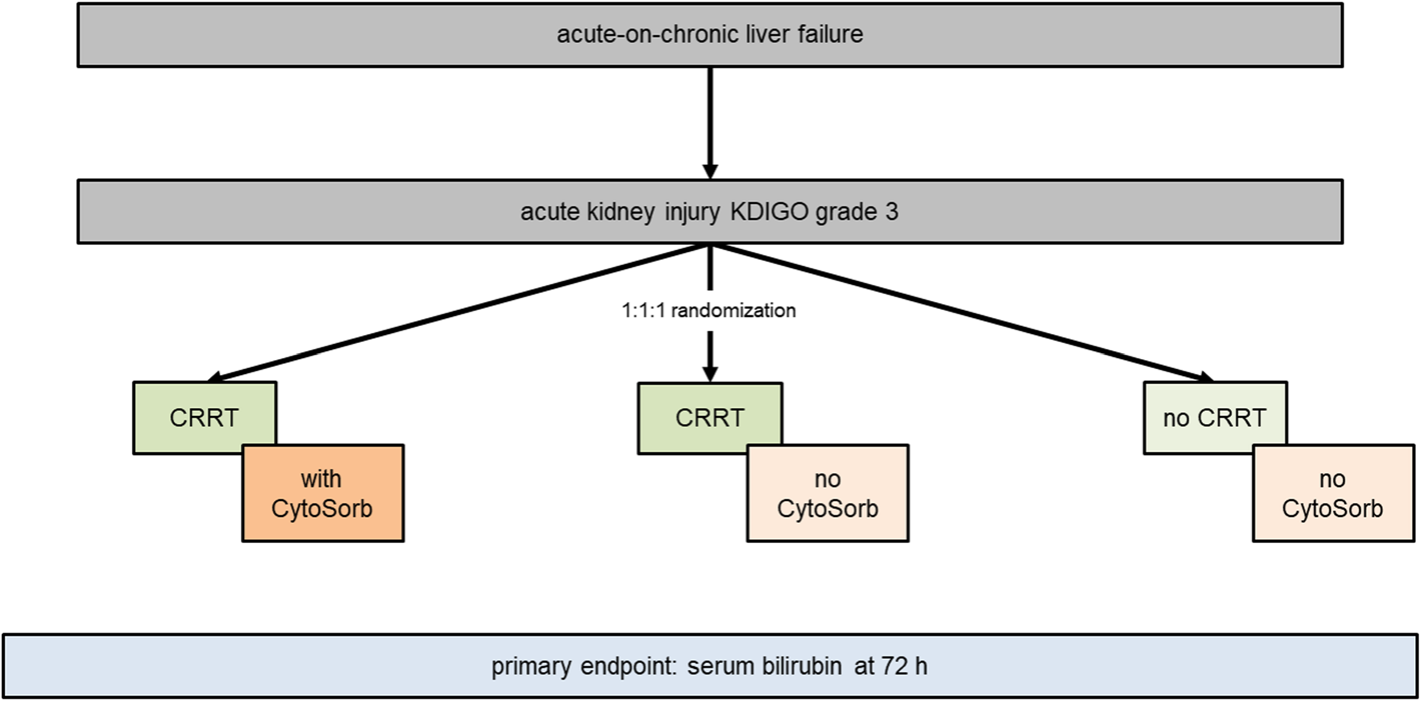
Graphical representation of the study design

The study is a prospective, randomized, open-label intervention study. Only patients with acute-on-chronic liver failure and AKI KDIGO stage 3 are included. In the CRRT with CytoSorb group, a CytoSorb adsorber is incorporated into the CRRT system for a total of 72 h. The adsorber is usually installed in the system as part of the preparation of the CRRT system before the system is connected to the patient circuit, but at the latest within 4 h after initiation of CRRT. The first adsorber can be left in the system for 12 h (± 6 h) and must then be replaced (*t*_12_). The replacement of the second adsorber will be 24 h after the start of adsorption therapy (*t*_24_). The third adsorber will be replaced at 48 h (*t*_48_) and the fourth adsorber will be removed at 72 h (*t*_72_) after the initial start of adsorption therapy. For the change adsorber 2 and 3 and the removal of adsorber 4 there will be a tolerance interval of ± 2 h for the respective time point (24 h, 48 h, and 72 h, respectively). Adsorber therapy will be applied for a total duration of 72 h using 4 adsorbers. In case of blood clotting in the adsorber and the CRRT circuit or any other reason for loss of function of CRRT with or without cytokine adsorption during the first 72 h, the CRRT system or the adsorber should be replaced as soon as possible. The therapy in the CRRT without the CytoSorb group differs from the intervention group only in that no CytoSorb adsorber is incorporated into the CRRT system. In the third group, patients received neither CRRT nor CytoSorb (Fig. 1).

**Fig. 2:**
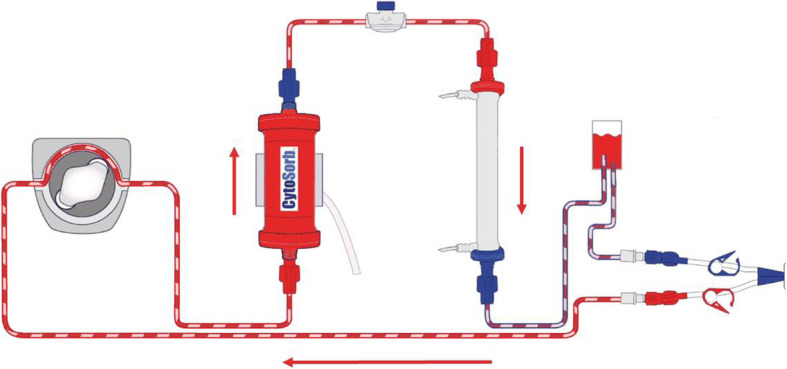
Schematic structure of the incorporation of the CytoSorb Adsorber into the CRRT system, blood flow in the direction of the arrows in red [source: https://cytosorb-therapy.com/de/der-adsorber/setup-von-cytosorb/]

Therapy of liver failure and potential concomitant complications of other organs will follow established standards and guidelines. In the CRRT with CytoSorb group, the CytoSorb adsorber will be connected pre-dialysis filter via a closed-loop circuit to the CRRT pump (multiFiltratePro, Fresenius Medical Care, see Fig. 2 for the setup). Recommended flow rates through the adsorber are between 100 ml/min and 700 ml/min. The choice of anticoagulation for the CRRT will be left to the discretion of the nephrologist on call. CRRT will be continued for at least 72 h and can then be stopped or continued by the discretion of the treating nephrologists or intensivists. CRRT dose can be calculated based on the effluent flow rate (= (dialysis flow in ml/hour + ultrafiltration rate in ml/hour)/body weight in kg). The recommended dose according to the KDIGO guideline is 20–25 mL/kg/h [22]. We will be aiming at approximately 25 mL/kg/h in order to achieve (despite interruptions and CRRT downtime, which are inevitable) a minimum effluent rate of 20 mL/kg/h over a 24-h period.

In addition to the routine diagnostics, additional blood samples are necessary within the scope of the study at 0 h/baseline/screening and 72 h (43.4 ml each). The reference time (*t*_0_) is the start of cytokine adsorption in the CRRT with the CytoSorb group and the start of CRRT in the CRRT without the CytoSorb group. In the no CRRT group, the reference time (*t*_0_) will be the time of randomization plus 2 h (since this is the expected average duration between randomization and initiation of CRRT in the CRRT groups).

The blood samples will be analyzed for standard clinical laboratory parameters, such as blood count, electrolytes, kidney and liver function parameters, and coagulation values. In addition, the levels of interleukins and cytokines will be determined, and peripheral blood mononuclear cells (PBMC) will be used for immunological analyses. The blood samples are taken via an inserted arterial catheter (A. radialis or A. brachialis) or a central venous catheter, which are necessary for regular therapy monitoring, drug treatment, and renal replacement therapy for the patients in the CRRT with CytoSorb and the CRRT without CytoSorb groups—no additional vascular punctures are required. Not all patients in the no CRRT group will require an arterial catheter or a central venous catheter. In these patients, blood draws are scheduled to be combined with routine blood draws necessary for treatment guidance, so that no additional vascular punctures are required.

The blood samples are analyzed in our hospital’s laboratories. Only a small proportion of the serum samples from each collection point are sent to an external laboratory (Olink Proteomics, Uppsala Science Park, SE-75183 Uppsala, Sweden) for analysis of further biomarkers. If sample residues remain after analysis, they will be discarded and disposed of properly. The samples are pseudonymized for dispatch, so that no conclusions can be drawn about individual patients in the external laboratory.

##### Criteria for discontinuing or modifying allocated interventions for a given trial participant

In case of harms or complications discovered during CRRT or cytokine adsorption, these treatments can be terminated or interrupted at any time. As in any extracorporeal circulation, blood clotting can occur and result in loss of function of the device. Continuous blood flow measurement in the CRRT system will allow early detection of clotting which will result in reduced blood flow. In this case, the adsorber or the CRRT Circuit may be exchanged at any time.

In case of any of the following conditions or situations, the study treatment for a patient will be terminated:

- Occurrence of a SA(D)E, A(D)E, or unanticipated (S)ADE, which, in the opinion of the investigator, indicates that continuation of the study treatment is not in the best interest of the subject
- At the discretion of the principal investigator or due to termination of the study by the principal investigator
- Inability to comply with the clinical trial protocol (CTP)

If the study treatment for a particular patient is terminated owing to an SA(D)E, A(D)E, or unanticipated (S)ADE, the patient will be observed until the event has either resolved or satisfactorily stabilized in the judgment of the principal investigator. The patient will remain in the study and all data continue to be recorded in the electronic case report form (eCRF) as described in the CTP.

##### Strategies to improve adherence to intervention protocols

Adherence to the intervention protocol depends primarily on the treating physicians. The study team including intensive care physicians, nephrologists, and hepatologists will be responsible for monitoring protocol adherence. Most importantly, study physicians will oversee timely exchange of the CytoSorb adsorbers and timely blood sample collections. Additional treatment of the patients will be independent from the study interventions and based on well-established treatment standards and standard operating procedures that are followed by the treating physicians.

##### Relevant concomitant care and interventions that are permitted or prohibited during the trial

Besides CRRT and cytokine adsorption in the CRRT with CytoSorb and the CRRT without CytoSorb groups and collection of study-associated additional blood samples, the treatment of the patients will follow established standards. There are no specific interventions that are prohibited during the trial.

#### Outcomes

##### Primary endpoint

The primary endpoint is serum bilirubin level after 72 h. The reference time (*t*_0_) is the start of cytokine adsorption in the CRRT with the CytoSorb group and the start of CRRT in the CRRT without the CytoSorb group. In the no CRRT group, the reference time (*t*_0_) will be the time of randomization plus 2 h (since this is the expected average duration between randomization and initiation of CRRT in the CRRT groups).

##### Secondary endpoints

- Survival time (days) from baseline
- Interleukin-6 after 72 h
- Liver function parameters (72 h): Quick/INR, AST, ALT, AP, g-GT
- Blood lactate (72 h)
- Clinical scores: CLIF-SOFA-score [38], MELD score [39], SOFA score [40], SAPS II [41], and FIPS score [42] (72 h)
- Ventilator-free days (VeFD) in the first 30 days after randomization, where each day on invasive mechanical ventilation (IMV), non-invasive ventilation (NIV), or ECMO is defined as ventilator day. VeFD=0 if the patient dies in the first 30 days after randomization
- Vasopressor free days (VaFD) in the first 30 days after randomization, where each day with any dose of epinephrine, norepinephrine, dobutamine, argipressin or terlipressin is defined as vasopressor day. VaFD=0 if the patient dies in the first 30 days after randomization
- Dialysis-free days (DFD) in the first 30 days after randomization, where each day on renal replacement therapy (RRT) is defined as dialysis day. DFD=0 if the patient dies in the first 30 days after randomization
- A biomarker panel of pro- and anti-inflammatory cytokines (blood samples will be frozen and stored for later analyses, the panel will be determined at the time of analysis)

Mean serum bilirubin levels and clinical scores at 72 h will be compared between treatment groups. Medians will be compared for other laboratory outcomes at 72 h. Survival times will be compared based on estimated hazard ratios and inspection of Kaplan-Meier curves. VeFD, VaFD, and DFD will be summarized by medians and compared between treatment arms based on the estimated probability of a better outcome (C-Index).

The primary endpoint and the secondary endpoints were selected for their relevance in the context of ACLF. Bilirubin is a core parameter describing disease severity and progression in ACLF. Bilirubin measurements are widely available. Previous retrospective analyses of patients with ACLF treated with CytoSorb described the course of bilirubin and these findings could therefore be used for the sample size estimation for this trial.

Interleukin-6 has previously been described as a prognostic factor in the context of the systemic inflammation hypothesis for ACLF. Liver function parameters, such as Quick/INR, AST, ALT, AP, g-GT are widely accepted routine parameters for the assessment of liver function, The CLIF-SOFA-Score, the MELD score, and the FIPS score are well-established scores for the assessment of liver disease severity. Blood lactate, VeFD, VaFD, and DFD describe disease severity and required treatment intensity for ICU patients. Finally, the SOFA score and SAPS II are well-established scoring systems for the assessment of disease severity of ICU patients.

##### Safety endpoints

Assessment of adverse (device) effects (A(D)E)), serious adverse (device) effects SA(D)E, unanticipated serious adverse device effects (USA(D)E)), device deficiencies (DD) and (serious) incidents during the study period. Proportions of patients with the respective incidents will be reported.

#### Participant timeline

**Fig. 3:**
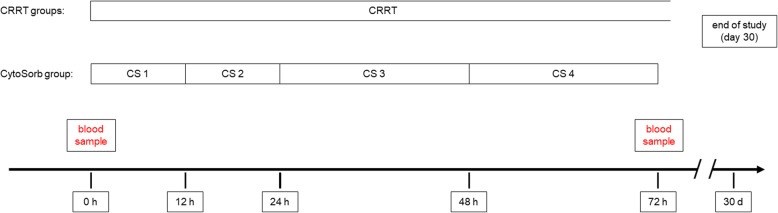
Participant timeline displaying timepoints of interventions and assessments during the 30-day study period. After the end of study (day 30) survival of the study participants will be followed up until day 90. CS, CytoSorb

The participant timeline is shown in Fig. 3.

#### Schedule of enrollment, interventions, and assessments

The schedule of enrollment, interventions, and assessments is shown in Table 1.

**Table 1:**
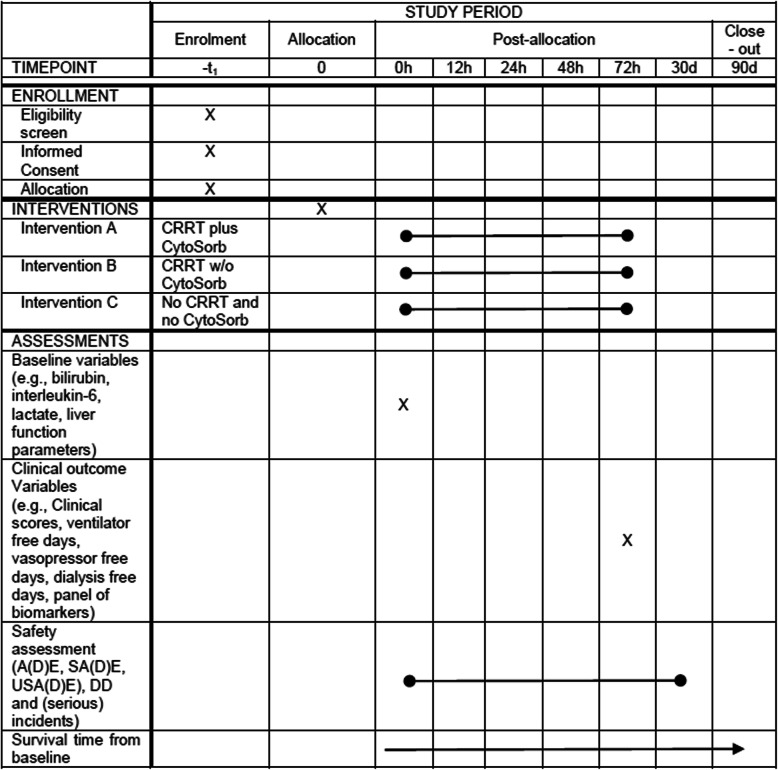
Schedule of enrollment, interventions, and assessments, tabular view

#### Sample size

In this study, the randomization to the treatment arms CRRT with CytoSorb, CRRT without CytoSorb, and no CRRT will be done with a 1:1:1 ratio. The group with no CRRT is included merely for exploratory analysis of safety aspects and does not affect the power analysis. The sample size calculation is motivated by data from case reports and retrospective case series, according to which serum bilirubin levels in patients with liver failure could be reduced by 50% by adsorption in the CytoSorb adsorber for 72 h [17–20]. We expect a mean of 10 mg/dl serum bilirubin at baseline, no change in control patients, and a reduction to a mean of 5 mg/dl after 72 h in the cytokine adsorption group. The typical range, 1 to 20 mg/dl, implies an expected standard deviation of about 5 mg/dl. This is a conservative estimate, as the inclusion criterion, bilirubin >5mg/dl, was not considered for the calculation. On the basis of a two-group *t*-test with a two-sided significance level of 5%, a sample size of 17 patients in each group yields a power of 80% to detect a difference if it is assumed that the mean serum bilirubin levels after 72 h differ by 5 mg/dl, given a standard deviation of 5 mg/dl in both groups. This is conservative in that we anticipate a lower standard deviation after 72 h in the intervention group. No missing values up to 72 h are expected. Hence, the numbers of patients to be randomized to the three treatment arms are *n*=17:17:17.

In the unexpected event that patients die within the study before reaching the 72-h end point, we will describe an appropriate expansion of enrolment in an amendment to this study protocol.

#### Recruitment

Patients with ACLF and AKI KDIGO stage 3 treated at the University of Freiburg Medical Center will be eligible for study participation. Patients meeting the inclusion criteria (or a legally authorized representative) will be asked for study participation and exclusion criteria will be assessed. All patients will be recruited by ASe, DB, or ASu. The first patient was recruited on December 7, 2021. Based on a retrospective analysis of patient records at our center, we expect about 50 patients per year meeting the inclusion criteria for this study.

### Assignment of interventions

#### Allocation

##### Sequence generation

Allocation to the intervention and control groups is carried out by 1:1:1 randomization, stratified for baseline serum bilirubin level (< 10 mg/dl, ≥ 10 mg/dl) and with blocks of variable length within strata. The block lengths will be documented separately and will not be disclosed to the investigators. Staff not otherwise involved in the trial will provide a computer-generated random list with the allocation sequence for the study groups.

##### Concealment mechanism

Allocation concealment will be ensured via an online randomization service (“The Randomizer”: www.randomizer.at). The random list will not be accessible to the clinical study team.

##### Implementation

When a patient with ACLF is selected for potential study inclusion a member of the study team will be informed. After assessment of inclusion and exclusion criteria informed consent will be obtained from the patient or from a legally authorized representative (see section 7.3. for details). Then a member of the study team will request online randomization according to the patient’s baseline bilirubin subgroup. When randomization results in “CRRT with CytoSorb” or “CRRT without CytoSorb” a CRRT machine will be ordered from the dialysis nurse on call and connected to the patient blood circuit, with or without a CytoSorb adsorber, respectively. If randomization results in “no CRRT” the patient will not receive early CRRT.

#### Blinding (masking)

The trial is an open-label trial, therefore, there is no blinding, neither for participants nor for the care providers or the study team.

### Data collection, management, and analysis

#### Data collection methods

##### Clinical data

Besides patient-specific demographic and personal data (e.g., age, height) medical pre-conditions, previous treatment in another hospital before referral to the Freiburg University Medical Center, and previous medication will be documented. Beyond this, during the study, clinical- and treatment-related data will be collected, including information on oxygen demand and ventilation parameters in patients requiring mechanical ventilation or high-flow oxygen support (see Table 2 for details) and clinical scores will be assessed at baseline and at 72 h (CLIF-SOFA-score [38], MELD score [39], SOFA score [40], SAPS II [41] and FIPS score [42]). Survival from baseline up to day 90 will be assessed for all patients. All clinical data will be retrieved from the electronic patient files (MEONA GmbH, Freiburg, Germany and COPRA System GmbH, Berlin, Germany). All clinical information collected for this study will be available from the electronic patient charts.

**Table 2:** Clinical data collected during the study. Data will be retrieved from the hospital’s electronic patient charts

	0 h	6 h	12 h	24 h	48 h	72 h	7 days
Supply of red cell concentrates [number]	x	x	x	x	x	x	x
Supply of thrombocyte concentrates [number]	x	x	x	x	x	x	x
Supply of fresh frozen plasma [number]	x	x	x	x	x	x	x
Dosage of norepinephrine [μg/kg/min]	x	x	x	x	x	x	x
Dosage of dobutamin [μg/kg/min]	x	x	x	x	x	x	x
Dosage of epinephrine [μg/kg/min]	x	x	x	x	x	x	x
Dosage of argipressin [IE/kg/min]	x	x	x	x	x	x	x
Dosage of terlipressin [mg/h]	x	x	x	x	x	x	x
Oxygen supply (l/min)	x	x	x	x	x	x	x
In patients requiring mechanical ventilation:							
FiO_2_	x	x	x	x	x	x	x
PEEP [mbar]	x	x	x	x	x	x	x
Body temperature	x	x	x	x	x	x	x
RRsys [mmHg]	x	x	x	x	x	x	x
RRdia [mmHg]	x	x	x	x	x	x	x
Mean arterial pressure [mmHg]	x	x	x	x	x	x	x
Heart rate [1/min]	x	x	x	x	x	x	x

##### Blood samples

Blood samples will be collected on the same way as blood samples in the clinical routine in our hospital. The samples will be analyzed in the hospital’s central laboratory. Since only parameters will be assessed that are available in clinical routine, no study-specific procedures need to be put in place. Table 3 lists the parameters that will be assessed.

**Table 3:** Laboratory parameters assessed at different time points during the study

	0 h	72 h	7 days*
Leukocytes [thous./μl]	x	x	x
Thrombocytes [thous./μl]	x	x	x
Red cell distribution with [%]	x	x	x
Hemoglobin [g/dl]	x	x	x
Hematocrit [%]	x	x	x
Lymphocytes [thous./μl]	x	x	x
Neutrophils [thous./μl]	x	x	x
Monocytes [thous./μl]	x	x	x
INR	x	x	x
PTT [sec.]	x	x	x
D-dimers [mg/l]	x	x	x
Fibrinogen [mg/dl]	x	x	x
Ferritin [ng/ml]	x	x	x
Urea [mg/dl]	x	x	x
Creatinine [mg/dl]	x	x	x
Uric acid [mg/dl]	x	x	x
LDH [U/l]	x	x	x
CK [U/l]	x	x	x
CK-MB [U/l]	x	x	x
Myoglobin [U/l]	x	x	x
Troponin T [ng/ml]	x	x	x
ProBNP [pg/ml]	x	x	x
AST [U/l]	x	x	x
ALT [U/l]	x	x	x
AP [U/l]	x	x	x
g-GT [U/l]	x	x	x
Bilirubin [mg/dl]	x	x	x
Bilirubin direct [mg/dl]	x	x	x
Bile acids [μmol/L]	x	x	x
CRP [mg/l]	x	x	x
PCT [ng/ml]	x	x	x
Protein [g/dl]	x	x	x
Albumin [g/dl]	x	x	x
IL-6 [pg/ml]	x	x	x
TNF-α	x	x	x
Triglycerides [mg/dl]	x	x	x
Cholesterine [mg/dl]	x	x	x
LDL-cholesterine [mg/dl]	x	x	x
HDL-cholesterine [mg/dl]	x	x	x
IgG [mg/dl]	x	x	x

*Parameters after 7 days will only be collected, if they are available within clinical routine. No additional blood samples will be taken

In all patients with CRRT, point-of-care blood gas analysis testing will be performed at least every 4–6 h as part of clinical routine assessments (available parameters: pH, pCO2 [mmHg], pO2 [mmHg], sO2 [%], SBC [mmol/l], Hb [g/dl], Na [mmol/l], K [mmol/l], Ca [mmol/l], Cl [mmol/l], lactate [mmol/l], glucose [mmol/l], bilirubin [mmol/l]). The results from these analyses, including serum bilirubin concentrations, will be available from the patient files and retrieved from the patient records for assessment in this trial. In the no CRRT group, these parameters will most likely not be available for all patients, since not all will require an arterial vascular access and repeated point-of-care blood gas analysis testing.

In addition to routine parameters and peripheral blood mononuclear cells (PBMC), small amounts of patient serum will be collected, frozen at – 80 °C and stored in a specifically designated refrigerator. After enrollment of all patients, all samples will then be sent to an external laboratory (Olink Proteomics, Uppsala Science Park, SE-75183 Uppsala, Sweden). If sample residues remain after analysis, they will be discarded and disposed of properly. The samples are anonymized for dispatch, so that no conclusions can be drawn about individual patients in the external laboratory.

##### Participant retention and follow-up

As described in the “Statistical methods” section, adherence to intervention protocols and participant retention primarily depend on the treating physicians. Therefore, no specific measures need to be taken to increase patient retention. Since no specific follow-up is planned in this study, no specific measures need to be taken in this respect, either.

##### List of clinical data and blood samples to be collected

List of clinical data and blood samples to be collected is shown in Tables 2 and 3.

#### Data management

Study data will be entered in the pseudonymized form in a study database by authorized and trained members of the study team via electronic case report forms (eCRF). The electronic data capture system REDCap^TM^ is used for data acquisition. This system uses built-in security features to prevent unauthorized access to patient data, including an encrypted transport protocol for data transmission from the clinical site to the study database. An audit trail provides a history of the data entered, changed, or deleted, indicating the processor and date. Access will be granted to authorized personnel only, and only if they have received appropriate training. The study database is located on a server of the IT facility (Klinikrechenzentrum, KRZ) of Medical Center - University of Freiburg. This server is expertly protected against access over several levels (see also the documentation of the IT facility).

The decision and control over the use of study data lie with the coordinating investigator. Compliance with relevant data protection regulations according to the EU-GDPR, federal and local data protection laws is guaranteed.

#### Statistical methods

##### Statistical methods for analyzing primary and secondary outcomes

Confirmatory statistical testing will be restricted to the analysis of the primary outcome, to be followed by descriptive reporting of other trial results**.** Emphasis will be given to reporting of effect estimators with two-sided 95% confidence intervals rather than *p* values, which will be considered statistically significant if below 5% for tests of equality.

In the primary analysis, randomized groups will be compared using a sequential closed test procedure: To guarantee a multiple significance level of 5%, confirmatory statistical testing at nominal significance level *α*=5% will continue until the first non-significant result, followed by descriptive reporting of the results of subsequent analyses. Treatment arms will be compared in the following order: CRRT with CytoSorb versus CRRT without CytoSorb, CRRT without CytoSorb versus no CRRT, CRRT with CytoSorb versus no CRRT.

The effects of CRRT with CytoSorb versus CRRT without CytoSorb with respect to the primary endpoint serum bilirubin level after 72 h will be estimated and tested within a linear regression model, and the two-sided 95% confidence interval will be calculated. The model will include randomized treatment group and continuous baseline bilirubin level as independent variables. Robust standard errors will be used to allow for different error variability in the two treatment groups. The two-sided test for a difference between the interventions CRRT with CytoSorb and CRRT without CytoSorb at significance level 5% will be based on the two-sided 95% confidence interval from the linear regression model. This is expected to increase the power compared to a t-test, due to adjustment for baseline bilirubin level. The same modeling approach will be employed for the comparisons of CRRT without CytoSorb versus no CRRT and CRRT with CytoSorb versus no CRRT, respectively.

No interim analysis will be performed for the primary endpoint. Before the end of the study and enrollment of all participants, no early confirmatory conclusion of the superior efficacy of the study intervention can be drawn.

The most relevant secondary endpoint is survival time from baseline since these data are planned to be used for the sample size calculation in a subsequent multi-center trial where survival time will serve as the primary endpoint. Due to the frequent application of cytokine adsorption in medical practice, a subsequent trial will be warranted even if the data of this trial will not show a benefit for cytokine adsorption. The effects of CRRT with CytoSorb versus CRRT without CytoSorb with respect to survival time will be estimated and tested by Cox regression. The regression model will include treatment and continuous baseline bilirubin level as independent variables. As an estimate of the effect size, the hazard ratio between the two treatment arms will be given with the corresponding asymptotic two-sided 95% confidence interval. Rates of patients surviving from baseline will be estimated for the three treatment arms using the Kaplan-Meier method.

Another important secondary aspect of the trial is the preparation of translational research into the mechanism of action induced by cytokine adsorption. To this end, an exploratory analysis of a broad array of clinical and inflammatory parameters with a potential role in ACLF will be performed. Statistical analysis will be designed to identify those parameters that are most clearly affected by cytokine adsorption. The primary focus will be on patients randomized to CRRT with CytoSorb and CRRT without CytoSorb. During initial data analysis, a pre-specified set of monotone transformations will be applied to the 72 h measurements of each parameter in the array, with the aim to reduce skewness. The skewness of the transformed values will be calculated for each parameter and each transformation. Among the transformations considered, the one yielding the mean skewness closest to zero, averaging skewness over parameters, will be selected and applied to both baseline and 72 h measurements of each parameter. For each parameter, the effects of CRRT with CytoSorb versus CRRT without CytoSorb with respect to the change from baseline (transformed value after 72 h minus transformed value at baseline) will be tested within a linear regression model including treatment and the transformed baseline value as independent variables. Robust standard errors will be used to allow for different error variability in the two treatment groups. The two-sided p-values based on these linear regression models will be adjusted for multiple testing using the Benjamini-Hochberg method to control the False Discovery Rate of parameters claimed to differ between treatments at the 5% level [43, 44].

Descriptive analyses of the remaining secondary endpoints will be performed with regression models or statistical tests as appropriate for the respective type of data.

For safety reasons, mortality will be closely monitored throughout the trial. Criteria for early termination of the study are listed and explained in the “Early termination of the study” section.

##### Methods for any additional analyses

Demographic and other baseline data (including disease characteristics) will be summarized descriptively by a randomized treatment arm. Numbers of complete and missing data (if any) will be shown. Relative frequencies will be shown as valid % (number of patients divided by the number of patients with non-missing values).

##### Definition of analysis population relating to protocol non-adherence (e.g., as randomized analysis), and any statistical methods to handle missing data (e.g., multiple imputation)

Efficacy analyses will be performed primarily in the full analysis set (FAS) according to the intention-to-treat (ITT) principle. This means that the patients will be analyzed in the treatment arms to which they were randomized, irrespective of whether they refused or discontinued the treatment or whether other protocol violations are revealed. Regarding the primary endpoint, however, cases of death within 72 h will be excluded from the analysis and reported descriptively. No other types of missing data are expected for the primary endpoint.

### Monitoring

#### Data monitoring

The study will be conducted as a single-center trial. Data monitoring will therefore be performed by the study team, headed by the principal investigator. Due to the small sample size and single-center setting, no data monitoring committee is required.

The study procedures and the collected data are continuously monitored and controlled by the study team. If there are any signs that patients in the intervention group are at risk from the study measures or premature evidence of the inferiority of the control group, the study can be interrupted at any time. Due to the small sample size, no interim analysis is planned.

#### Harms

Unexpected complications will be monitored. Since the CytoSorb adsorber is a tested and approved medical device, such hazards are not to be expected. See the “Early termination of the study” section on provisions for early termination of the study due to safety concerns.

#### Auditing

In addition to the above-described monitoring activities, no specific auditing is required for this single-center trial.

## Ethics and dissemination

### Research ethics approval

This study protocol is approved by the University of Freiburg’s Ethics Committee (EK-FR 21-1470).

### Protocol amendments

All relevant protocol modifications will be submitted to the University of Freiburg’s Ethics Committee for approval. No changes will be implemented before ethical approval.

### Consent or assent

Before being enrolled in the study, written consent will be obtained either from the patient, a relative, or from a legally authorized representative (LAR).

Patients with ACLF eligible for study inclusion may suffer from impaired cognitive capacity, among others, owing to hepatic encephalopathy or acute renal failure. Most patients eligible for inclusion in the CYTOHEP study will not be able to adequately understand the current study and the study interventions, therefore, informed consent must be obtained from a relative or a LAR. When a patient with impaired cognitive capacity is admitted to the Freiburg University Medical Center, as part of a clinical routine and independent from any potential study participation, the medical care team (doctors and nursing staff) will ensure rapid contact with the patient’s relatives or a LAR. In general, relatives can be identified and contacted quickly. If these initial attempts are unsuccessful, the police may attempt to contact them. If the patient has no relatives or they cannot be identified or contacted, a request to appoint a LAR will be made to the local court as soon as possible. The LAR is then informed about the current research project. After regaining consciousness and the ability to give consent before the end of the examination period, the patient is informed again (Table 4).

Table 4 Variants of information on participation in the study

### Confidentiality

The name of the patients is not saved. However, the evaluation of age, sex, and time of admission merely provides a pseudonymization. Pseudonymization of personal data makes it much more difficult to identify the persons concerned and can only be done safely by using a list in which the pseudonym and patient name are combined. Pseudonymization is carried out directly in the raw data before any further processing, so that only the pseudonyms are recognizable in all evaluations. The decryption table is kept separately and protected; only the coordinating investigator and his deputies have access to this list. Participant´s study information will not be released outside of the study without the written permission of the participant, except as necessary for monitoring.

### Declaration of interests

Alexander Supady received speakers’ honoraria from CytoSorbents, the manufacturer of the CytoSorb device. Alexander Supady also received an unrestricted research grant from CytoSorbents. Dominik Bettinger received speakers’ honoraria from Bayer Healthcare and the Falk Foundation. Further, he receives consulting honoraria from Bayer Healthcare, Boston Scientific, and Shionogi. Eric Prager received consulting honoraria from Novartis Pharma. Erika Graf receives consultant fees from Roche Pharma AG.

### Access to data

All members of the study team and all researchers involved in data collection, evaluation, and writing of the scientific publication(s) related to this study will have full access to all data collected. There are no contractual agreements with anyone that would limit the access to the data by any means.

### Ancillary and post-trial care

Since there are no harms to be expected resulting specifically from the trial-specific interventions, no special provisions are made for trial-specific ancillary or post-trial care. In this study, CRRT and the Cytosorb adsorber are used within the scope of the intended purpose of the medical products with CE marking. The Cytosorb adsorber is used in the study under the same conditions as in clinical routine, so that no other hazards or risks occur and therefore, according to the MPG (Medizinproduktegesetz), there is no obligation for a specific insurance. The same is true for renal replacement therapy which is used according to established standards as in clinical routine. The additional blood samples in the study are taken via arterial or venous catheters, which are also inserted independently of the study participation, and do not represent a relevant risk; the total amount of blood collected is low. Patients are covered by the hospital’s regular liability insurance. Not all patients randomized to the safety control group without CRRT will require an arterial catheter or a central venous catheter. In these patients, blood samples are taken via peripheral venous punctures. The risk of these additional punctures can be considered very low and they are necessary for guiding treatment in these patients independent of their participation in the trial.

### Early termination of the study

The study may be discontinued at the discretion of the principal investigator or if any of the following criteria are met:

- Medical or ethical concerns
- The safety of the participants is doubtful or at risk, respectively
- Too many AEs unknown previously (i.e., not previously reported in any similar study or in market use with respect to their nature, severity, and/or duration)
- Alterations in accepted clinical practice that make the continuation of a clinical trial unwise
- Early evidence of benefit or harm of the experimental intervention
- Difficulties in the recruitment of patients

In a recent randomized controlled pilot trial in 34 COVID-19 patients on ECMO (CYCOV study), an association between cytokine adsorption and increased mortality has been described [32]. With specific respect to these findings, all patient deaths within the CYTOHEP study will be evaluated continuously and before inclusion of any further patient into the trial, the risk-benefit ratio for continuation of the study will be assessed by the principal investigator. After inclusion of every fifth patient, the principal investigator, the co-principal investigators, and the trial statistician will discuss the risk-benefit ratio for continuation of the study based on the results of the patients included until then and come to a joint decision as to continue or discontinue the study.

Optimal timing of initiation of CRRT in AKI is a matter of ongoing debate. In the CRRT groups of this trial, CRRT will be initiated when patients reach AKI KDIGO grade 3. However, in our hospital, CRRT in AKI in general is initiated later, i.e., not before patients develop life-threatening complications, such as severe hyperkalemia or severe respiratory failure due to volume overload. In patients with ACLF, specifically, there is no clear evidence supporting an early or a late initiation strategy for CRRT. For safety reasons, we will include a third study group without CRRT as a safety control group. Patients randomized to this group will not receive early CRRT as in the other groups but the use of CRRT will be delayed until the development of one or more of the following criteria: a serum potassium level of 6.0 mmol or more per liter, a pH of 7.20 or less due to metabolic acidosis or a serum bicarbonate level of 12 mmol per liter or less, evidence of severe respiratory failure based on a ratio of the partial pressure of arterial oxygen to the fraction of inspired oxygen of 200 or less and clinical perception of volume overload. We will continuously monitor survival, complications, and safety of the treatment in all study groups to be able to detect early any potential harm related to CytoSorb treatment or early initiation of CRRT in comparison to the no CRRT group.

### Dissemination policy

The results of this study will be presented at symposia and conferences and published in scientific journals. Assistance of a professional writer for the scientific publication(s) is not intended but will not generally be rejected. Authorship eligibility will follow the recommendation provided by the International Committee of Medical Journal Editors:

http://www.icmje.org/recommendations/browse/roles-and-responsibilities/defining-the-role-of-authors-and-contributors.html

After completion of the trial, de-identified individual participant data that underlie the results reported in the publication of this trial will be made available for individual participant data meta-analysis to researchers who provide a methodologically sound proposal beginning 9 months and ending 36 months following article publication, if in compliance with data protection legislation. Proposals should be directed to the corresponding author; to gain access, data requestors will need to sign a data access agreement.

## Discussion

The randomized, controlled CYTOHEP study is designed to evaluate the use of extracorporeal hemoadsorption using the CytoSorb device in patients with acute-on-chronic liver failure. Even though the CytoSorb device is frequently being used for the treatment of patients with liver failure, there is only limited evidence supporting this approach. Pre-clinical studies and retrospective patient data analyses suggest technical efficiency of the CytoSorb in reducing elevated levels of bilirubin and IL-6. Both parameters occur in excess in the patients’ blood in acutely decompensated liver cirrhosis. However, currently, there is no reliable evidence about the effect on patient-centered outcomes, including survival. The aim of the CYTOHEP study is to close this gap and provide evidence for or against the use of hemoadsorption with the CytoSorb device for these patients. To assess a potential effect of renal replacement therapy on the outcome of the patients, we also included a third group into this trial not receiving renal replacement therapy or CytoSorb. The controlled design of this study will allow to independently assess the effect of CytoSorb and renal replacement therapy on laboratory parameters and patient outcome. The single-center design will guarantee comparable standards for ICU treatment in all three arms of this trial.

## Data Availability

For the planning of this study and writing of the manuscript no specific primary data was collected. After completion of the trial, de-identified individual participant data that underlie the results reported in the publication of this trial will be made available for individual participant data meta-analysis to researchers who provide a methodologically sound proposal beginning 9 months and ending 36 months following article publication, if in compliance with data protection legislation. Proposals should be directed to the corresponding author; to gain access, data requestors will need to sign a data access agreement.

## Abbreviations

A.: Arteria
ACLF: Acute-on-chronic liver failure
A(D)E: Adverse (device) effect
AKI: Acute kidney injury
ALT: Alanine aminotransferase
AP: Alkaline phosphatase
AST: Aspartate aminotransferase
BUN: Blood urea nitrogen
Ca: Calcium
CE: Conformité Européenne
CK: Creatine kinase
CK-MB: MB-isoenzyme of creatine kinase
Cl: Chloride
CLIF-SOFA: Chronic liver failure sequential organ failure assessment
COVID-19: Coronavirus disease 2019
CS: CytoSorb
CTP: Clinical trial protocol
CYCOV: Cytokine adsorption in patients with severe COVID-19 pneumonia requiring extracorporeal membrane oxygenation
CYTOHEP: Cytokine adsorption in patients with acute-on-chronic liver failure
CRRT: Continuous renal replacement therapy
CRP: C-reactive protein
DAMP: Damage-associated molecular patterns
DD: Device deficiency
DFD: Dialysis free days
DRKS: Deutsches Register Klinischer Studien
ECMO: Extracorporeal membrane oxygenation
eCRF: Electronic case report form
EU-GDPR: European Union General Data Protection Regulation
FAS: Full Analysis Set
FiO_2_: Fraction of inspired oxygen
FIPS: Freiburg index of post-TIPS survival
g-GT: Gamma glutamyl transferase
Hb: Hemoglobin
ICU: Intensive care unit
IgG: Immunoglobulin G
IL-6: Interleukin-6
IMV: Invasive mechanical ventilation
INR: International Normalized Ratio
IRB: Institutional Review Board
ITT: Intention to treat
K: Potassium
KDIGO: Kidney Disease: Improving Global Outcome
KRZ: Klinikrechenzentrum
LAR: Legally authorized representative
LDH: Lactate-dehydrogenase
MARS: Molecule adsorbent recirculating system
MELD: Model of end-stage liver disease
MPG: Medizinproduktegesetz
Na: Sodium
NIV: Non-invasive ventilation
PAMP: Pathogen-associated molecular patterns
PBMC: Peripheral blood mononuclear cells
PaCO_2_: Partial pressure of carbon dioxide
PaO_2_: Partial pressure of oxygen
PCT: Procalcitonin
PEEP: Positive end-expiratory pressure
pro-BNP: Pro-brain natriuretic peptide
PTT: Partial thromboplastin time
RCT: Randomized controlled trial
RRdia: Diastolic blood pressure
RRsys: Systolic blood pressure
RRT: Renal replacement therapy
SA(D)E: Serious adverse (device) effect
SAPS II: Simplified acute physiology score II
SBC: Serum bicarbonate
SOC: Standard of care
SOFA: Sequential organ failure assessment
TNF-α: Tumor necrosis factor alpha
USA(D)E: Unanticipated serious adverse device effects
VaFD: Vasopressor free day
VeFD: Ventilator free day
WHO: World Health Organization
