# Correction: Cytokine adsorption in patients with acute-on-chronic liver failure (CYTOHEP)—a single center, open-label, three-arm, randomized, controlled intervention trial

**DOI:** 10.1186/s13063-022-06267-z

**Published:** 2022-04-15

**Authors:** Asieb Sekandarzad, Enya Weber, Eric Peter Prager, Erika Graf, Dominik Bettinger, Tobias Wengenmayer, Alexander Supady

**Affiliations:** 1 Department of Medicine III (Interdisciplinary Medical Intensive Care), Medical Center, University of Freiburg, Faculty of Medicine, University of Freiburg, Freiburg, Germany; 2 Department of Cardiology and Angiology I, Heart Center, University of Freiburg, Freiburg, Germany; 3 Institute of Medical Biometry and Statistics, Faculty of Medicine and Medical Center, University of Freiburg, Freiburg, Germany; 4 Department of Medicine IV (Nephrology and General Medicine), Medical Center, University of Freiburg, Faculty of Medicine, University of Freiburg, Freiburg, Germany; 5 Department of Medicine II (Gastroenterology), Medical Center, University of Freiburg, Faculty of Medicine, University of Freiburg, Freiburg, Germany; 6 Heidelberg Institute of Global Health, University of Heidelberg, Heidelberg, Germany

**Correction to: Trials 23, 222 (2021)**

**https://doi.org/10.1186/s13063-022-06139-6**

Following the publication of the original article [1], we were notified that Table 4 was mentioned in the article, but was not displayed.

The original article has been corrected to include Table 4.
